# Validity and reliability test for problem areas in Diabetes-Five item short form (PAID-5) Indonesia version

**DOI:** 10.12669/pjms.39.3.7088

**Published:** 2023

**Authors:** Tunjung Arini Budi, Raden Bowo Pramono, Anggi Lukman Wicaksana

**Affiliations:** 1 Tunjung Arini Budi, BN, RN. Student, School of Nursing, Universitas Gadjah Mada, Yogyakarta, Indonesia; 2 Raden Bowo Pramono, MD. Endocrinologist Consultant, Assistant Professor, Department of Internal Medicine,Universitas Gadjah Mada, Yogyakarta, Indonesia. Consultant Endocrinology, Department of Internal Medicine, Dr. Sardjito General Hospital, Yogyakarta, Indonesia; 3 Anggi Lukman Wicaksana, MS, BN, RN. Assistant Professor, Department of Medical Surgical Nursing, Universitas Gadjah Mada, Yogyakarta, Indonesia. Researcher, The Sleman Health & Demographic Surveillance System, Universitas Gadjah Mada, Yogyakarta, Indonesia. PhD Student, School of Nursing, College of Nursing, Taipei Medical University, Taiwan, ROC

**Keywords:** Diabetes mellitus, Emotional distress, Indonesian version, PAID-5, Psychometric testing, Reliability, Validity

## Abstract

**Background and Objective:**

Emotional distress experienced by patients with diabetes (PWD) can affect glycemic control and quality of life. However, limited tools are available in Indonesia to detect emotional distress in PWD in clinical setting or research. This study aimed to evaluate the validity and reliability of the Indonesia version of the Problem Areas in Diabetes (PAID-5) scale.

**Methods:**

After the cross-cultural adaptation method was conducted, psychometric tests were done from August to November 2019 at affiliated hospitals in Yogyakarta by involving 100 adult PWD. All PWD with no medical records of mental health problems or cognitive disorders were voluntarily included. Content and construct validity and internal consistency measurements were used to evaluate the psychometric properties.

**Results:**

The mean age was 61.2 years of the men and women who equally participated in the study and mostly were non-working patients. The PAID-5 resulted in five question items to identify the emotional distress of PWD in the Indonesian language. Some minor modifications were done in items four and five after discussing them with the original authors and experts in Indonesia. The results showed item content validity index for item and scale were 0.6-0.8 and 0.72, respectively. The calculated r-values ranged from 0.751 to 0.888, which were higher than the r table (0.197). The Cronbach alpha of the Indonesia version of PAID-5 was 0.87 with inter-item and item-total correlations of 0.43-0.71 and 0.61-0.79, respectively.

**Conclusion:**

The results of the study indicate that PAID-5 is considered valid and reliable to assess emotional distress among PWD and can be useful in clinical setting or for research purposes. Continued assessment of emotional distress is applicable and helps patients to better deal with their emotional distress.

## INTRODUCTION

Diabetes is reported to be among the top 10 causes of death globally. In 2017, the number of patients with diabetes (PWD) in Indonesia was ranked 6th in the world with the prevalence of 10.3 million. In 2045, the estimated prevalence will increase to about 16.7 million.^1^ Diabetes management and unachieved diabetes control could result in emotional distress among PWD.^2^,^3^ More than 44% of PWD were noted experiencing emotional distress due to diabetes.^4^ These problems lead to non-medication adherence, unachieved glycemic controls, and poor quality of life among diabetic patients.^5^-^7^ Accordingly, the identification of emotional distress can help to minimize the diabetes complications and improve the well-being of PWD.^8^,^9^

The emotional distress assessment is a necessary part of the comprehensive approach to treatment interventions during medical visits.^10^ This evaluation is also a basic need in diabetes management because the clinical incidence of the medical problems-related psychological disease is more frequent among diabetic patients than those without diabetes.^3^ *A valid, reliable, and brief evaluation tool is required to assess emotional distress for PWD. The Problem Areas in Diabetes (PAID)* is known as a specific instrument to measure emotional distress among patients with type-1 and type-2 diabetes, originally consisting of 20 items. It is applicable either in clinical settings or for research-based purposes.^11^ The PAID-5 is a shorter form of the original version and has only five items. It is mostly usable in clinical settings for rapid screening of diabetes-related emotional distress.^12^ A rapid, but valid tool such as PAID-5 is needed in the limited time available in clinics.^13^,^14^ It could help to identify PWD with high risk for emotional distress in a few minutes.

Several versions of translated PAID-5 are available. The Korean and Norwegian versions exist with valid and reliable outcomes.^15^,^16^ To the authors’ best knowledge, there is currently no available PAID-5 version in Indonesia. Therefore, to make the regular and rapid evaluation of emotional distress in Indonesia, it is important to have the PAID-5, a version in Indonesian language. This study aimed to evaluate the PAID-5 and to test its reliability and validity among PWD in determining their emotional distress.

## METHODS

All research processes were approved by the Medical and Health Research Ethics Committee (Ref: KE/FK/0511/EC/2019, 7 May 2019). Each participant received a printed version with an explanation of the study as well as verbal instructions. Their involvement in the study was voluntary and they had the full right to withdraw from the study at any time. To maintain their privacy, no identification information was required and they were asked to choose the ideal meeting situation.

A cross-sectional design was used for psychometric testing and data were collected in August to November 2019 at affiliated hospitals in Yogyakarta. Patients were included using the consecutive sampling technique based on daily registration lists. Considering the minimum 20 participants for each item in the questionnaire,^17^ a minimum of 100 participants should be included in this research. Adult PWD who visited a diabetes clinic, understand the Indonesian language, no medical record or history of mental illness and cognitive impairment from the electronic health records in the hospital, able to actively participate, were asked to join the study. Those who were eligible and agreed to participate were asked to sign the informed consent form and follow the procedures during data collection.^11^

The cross-cultural adaptation of PAID-5 was done before the data collection stage. The PAID-5 was specifically developed to detect emotional distress in patients with type-1 and type-2 diabetes and is an existing valid instrument with a brief version. The PAID-5 consists of five items, which are rated on a 5-point Likert scale from 0-4 (zero = not a problem; one = minor problem; two = moderate problem; three = somewhat serious problem; and four = serious problem). All items are added together and the total score ≥ eight indicates emotional distress. The possible total score of PAID-5 ranges from 0 to 20. The greater obtained score indicates the greater experience of emotional distress.^18^ This instrument was translated into the Indonesian language with permission from the original authors of PAID-5. The following process was performed: forward translation (by two bilingual speakers of Indonesian and English who translated it into the Indonesian language), synthesis (the researchers assessed the consistency between the original and translated versions), backward translation (by two bilingual professional sworn translators who translated them back into English), and an expert panel review (five multidisciplinary experts of diabetes care judged and provided feedback).^3^,^17^ This process resulted in the pre-final version of PAID-5 and content validity index (CVI) scores from the experts. The expert panel consisted of members of the nursing faculty, an endocrinologist, diabetes nurses, and a psychologist, with minimum five years expertise in the field of diabetes care who reviewed the Indonesian and English versions. It was recommended to invite a minimum of three to ten multidisciplinary experts in a panel for content analysis.^18^ The panel of experts evaluated the items for relevancy, using a 4-point Likert scale (one = not relevant, two = somehow relevant, three = quite relevant, and four = highly relevant) and clarity, readability and equivalence using the same pattern (one = not clear, two = somehow clear, three = clear, and four = very clear).^13^,^17^

As a part of content validity testing, the CVI for item (I-CVI) and scale (S-CVI) were calculated. In addition, the experts could give feedback about the questions which were discussed during the expert panel review with the authors. Following the step, a pilot testing of the pre-final version was tested on ten type-2 diabetes patients to see the feasibility of PAID-5. The study was continued through testing of the final version of PAID-5 to the eligible patients in several diabetes clinics at affiliated hospitals. This step was aimed to assess the construct validity and internal consistency. Nurses in charge assisted in the identification of potential participants using their list of diabetic patients through consecutive sampling technique. The medical records confirmed the criteria and the researchers approached the patients to explain the study to them during waiting for their routine visit. The full set of patient information Form and self-administered questionnaire was provided to each participant including demographic characteristics (i.e. age, sex, level of education, employment status, income status, and marital status). The researchers accompanied the participants while they completed it then checked the questionnaire for completion. The completed questionnaire was stored in a closed envelope after self-completion by each of the participants.^11^

Data analyses were done using Statistical Package for Social Science-SPSS version 22 (IBM Corp., Armonk, NY, USA). The univariate analysis was used to summarize the participant characteristics such as age, sex, level of education, employment status, income status, and marital status. Moreover, the Polit and Back model was used to evaluate the I-CVI and S-CVI. The criterion of an adequate item of CVI was more than or equal to 0.78 and more than or equal to 0.80 for subscale of CVI.^13^,^18^ Furthermore, the construct validity was analyzed using Pearson’s product–moment correlation. The item was considered valid when r-value is greater than the r table (r table = 0.197; N = 100, α = 0.05).^19^ The reliability test used internal consistency with Cronbach’s alpha coefficient as the main outcome. The Cronbach’s alpha >0.80 indicates highly reliable.^20^ Inter-item correlation between 0.30-0.70 indicates adequate and acceptable coefficients, while item-total correlation which is higher than 0.30 indicated an acceptable coefficient.^18^

## RESULTS

A total of 100 patients with type-2 diabetes participated in the study (response rate 100%). The mean age of the participants was 61.2 years (standard deviation = 9.6 year), ranging from 39-82 years. Men and women had equal distribution by chance (50%), with one-third of them having a diploma/bachelor degree (37%), and who mostly were non-working/retired (74%, Table-I).

**Table-I:** Demographic characteristics of participants (n = 100).

Demographic characteristic	n/%	Demographic characteristic	n/%
Age, Mean ± SD	61.2 ± 9.6	Monthly Income*	
Gender		<1 million	38
Woman	50	1-< 2 million	10
Man	50	2-< 3 million	8
Education level		3-< 4 million	19
No education	3	4-< 5 million	13
Primary school	12	> 5 million	12
Middle school	13	Marital status	
High school	27	Married	82
Diploma/bachelor	37	Single/divorced	18
Magister/doctoral	8		
Employment status			
Employed	26		
Unemployed/retired	74		

***Note:***

* in Indonesian Rupiah; SD = standard deviation.

The results of the pilot testing on 10 participants showed 1 of the 5 items (item five) was difficult to understand, while 80% of participants declared that the instrument was easy to understand. In total, they required about one minute and four seconds to complete it.

An expert panel evaluated the content validity of PAID-5. The clarity, readability and equivalence of the PAID-5 were 84%, 84%, and 88%, respectively, indicating a promising outcome. The I-CVI ranged between 0.60 and 0.80. The S-CVI score was 0.72. Two items (items 4 and 5) had I-CVI below 0.70. Accordingly, some modifications were done after discussing the original content of the items together with the research team and the experts. One word in item 4, “power” was modified into “energy” (synonym). For item 5, the statement of “facing complication of diabetes” was modified to “ability to face complications of diabetes” (making it clearer and straightforward). This change corresponded with the pilot result in which participants had some difficulty to understand the content of item 5.

The construct validity analysis used a comparison between r-value and r-table. The R-values ranged between 0.751-0.888 (r-table = 0.197; N = 100, α = 0.05, Table-II). All items indicated a higher value than the comparator in r-table.

**Table-II:** Pearson’s correlation test of PAID-5 Indonesian Version (N = 100).

Number of Item	r-value	Interpretation
1	0.751	VALID
2	0.810	VALID
3	0.888	VALID
4	0.789	VALID
5	0.808	VALID

The Cronbach’s alpha coefficient of PAID-5 was 0.87. The inter-item correlation resulted in a range between 0.43-0.71, whereas the item-total correlation resulted in a range between 0.61-0.79 (Table-III).

**Table-III:** Internal consistency with Cronbach’s alpha of PAID-5 Indonesia Version.

Number of Item	Inter-item Correlation	Item-total Correlation	Cronbach’s alpha if item deleted
1	0.43-0.58	0.618	0.856
2	0.55-0.71	0.714	0.836
3	0.58-0.71	0.793	0.812
4	0.43-0.59	0.656	0.848
5	0.47-0.68	0.693	0.838

## DISCUSSION

This current study assessed the psychometric properties of the short form of PAID-5 Indonesia Version among patients with type-2 diabetes. The results showed acceptable outcomes of validity and reliability for the PAID-5 Indonesia Version. The cross-cultural adaptation process of PAID-5 was similar with the Korean and Norwegian versions.^15^,^16^ Content and construct validity, as well as internal consistency for reliability measurements were tested.

The clarity, readability, and equivalence of the PAID-5 indicated satisfactory results. All items in the PAID-5 were found reflecting the intended meaning from the original version, and were considered unambiguous and understandable. I-CVI scores ranged between 0.6-0.8 and S-CVI was 0.72. Items with an I-CVI of 0.70 or greater were retained, while items between 0.5-0.7 were modified or revised.^21^ Items 4 and 5 had I-CVI scores lower than 0.70 and underwent some modifications based on the experts’ comments. In addition, the score of S-CVI was lower than 0.8. Therefore, minor modification was required on the specific items based on the experts’ comments, supporting the I-CVI findings.^19^,^22^ This modification process was done by considering the original concept of the item and the experts’ comments. The researchers facilitated a discussion with the research team members and experts. As a result, per the consent of the experts, the modification used a synonym in item 4 and a more complete statement in item 5. Modification of the language in items is common during the cross-cultural adaptation process to suit the specific population or society without losing their own meaning.^3^

Most participants declared that the PAID-5 instrument was easy to understand and required only about one minute to complete. This finding was similar with the original version which only needed less than one minute to complete.^12^ A single minute is a relatively short time for filling in a questionnaire and makes the PAID-5 more applicable in clinical settings where there is limited available time. The short time of filling in the questionnaire also makes the regular or rapid screening feasible.^13^,^14^

The r-value of PAID-5 ranged between 0.751-0.888 and it was greater than r-table (0.197), which indicated that all of the items on PAID-5 were valid. Coefficient correlation (r) was categorized as 0.9-1.0 indicating very highly correlated, and 0.7-0.9 indicating highly correlated.^23^ Results showed that PAID-5 was highly correlated, indicating the significant relationship among items and related each of them to the emotional distress construct. Similar constructs will have the similar pattern which focused on the same variable and have significant correlation. These findings were relatively higher than the Korean version (0.65-0.84) and the Norwegian version of PAID-5 (0.72-0.85) among PWD.^15^,^16^

The score of Cronbach’s alphas of PAID-5 was 0.87, showing a highly reliable outcome. This score indicated that the PAID-5 was consistent in measuring emotional distress.^21^ Inter-item correlation and item-total correlation ranged between 0.43-0.71 and 0.61-0.79, respectively. Inter-item correlation ranges between 0.30 and 0.70 show adequate and acceptable results, while item-total correlations greater than 0.30 show acceptable results.^19^,^24^-^26^ The results showed that this instrument was highly reliable, adequate, and acceptable to assess emotional distress among PWD. The result of the Cronbach’s alpha in the PAID-5 was similar to the Korean version (0.87)^15^ and slightly lower than the Norwegian version (0.89).^16^ The Norwegian version of PAID-5 reported inter-item correlations that ranged from 0.49 to 0.74. These findings were closely and relatively similar with our findings for PAID-5.^16^ Thus, the reliability outcomes of PAID-5 indicated it is reliable and consistent to measure emotional distress.

To the best of our knowledge, this paper provided the first information about the validity and reliability of the PAID-5 Indonesian version. This evidence supports the early detection of emotional distress among PWD, even as early as pre-school children.^27^ These findings are remarkably crucial for future research and clinical practice. Further research related to quality of life and healthy lifestyles of PWD for both adults and children may apply this tool to measure or identify the emotional distress among PWD and to recommend appropriate treatment programs, including healthy diet and regular physical activity.^28^,^29^ Furthermore, clinicians could collect valid data of emotional distress within two minutes to assist them in treating the health problems in diabetic patients.

### Clinical significance of study:

The study provides information about the validity and reliability of PAID-5. The results ensure that the PAID-5 is applicable to assess and evaluate the emotional distress among PWD. The tool is brief and requires short time (one-two minutes) thus it is useful for clinical setting where there is limited available time. By detecting the emotional distress among PWD, clinicians can do early treatment and management before the patient develops more serious problems.

### Limitations:

The study was conducted in a tertiary clinic that receives referral patients from several hospitals to enlist more diabetic patients who are experiencing emotional distress. However, they only covered type-2 diabetes patients. The original version of PAID-5 is also applicable for type-1 diabetes. Further analysis and study for type-1 and gestational diabetes may be required to see the similar evidence of psychometric properties.

## CONCLUSIONS

The PAID-5, an Indonesia Version of PAID-5, can be considered as a valid and reliable instrument to measure emotional distress among patient with diabetes. The results of this psychometric testing confirmed the validity and reliability of the PAID-5 Indonesian version in identifying the emotional distress among patients with diabetes. Therefore, this tool may be applicable in terms of research or clinical purposes.
